# Predicting outcome following psychological therapy in IAPT (PROMPT): a naturalistic project protocol

**DOI:** 10.1186/1471-244X-14-170

**Published:** 2014-06-09

**Authors:** Nina Grant, Matthew Hotopf, Gerome Breen, Anthony Cleare, Nick Grey, Nilay Hepgul, Sinead King, Paul Moran, Carmine M Pariante, Janet Wingrove, Allan H Young, André Tylee

**Affiliations:** 1 Department of Psychology, Institute of Psychiatry, King’s College London, London, UK; 2 Department of Psychological Medicine, Institute of Psychiatry, King’s College London, London, UK; 3 MRC Social, Genetic, and Developmental Psychiatry Centre, Institute of Psychiatry, King’s College London, London, UK; 4 Centre for Anxiety Disorders and Trauma, South London & Maudsley NHS Foundation Trust, London, UK; 5 Health Services and Population Research, Institute of Psychiatry, King’s College London, London, UK; 6 Southwark Psychological Therapies Service, South London & Maudsley NHS Foundation Trust, London, UK; 7 Centre for Affective Disorders, Institute of Psychiatry, King’s College London, London, UK

## Abstract

**Background:**

Depression and anxiety are highly prevalent and represent a significant and well described public health burden. Whilst first line psychological treatments are effective for nearly half of attenders, there remain a substantial number of patients who do not benefit. The main objective of the present project is to establish an infrastructure platform for the identification of factors that predict lack of response to psychological treatment for depression and anxiety, in order to better target treatments as well as to support translational and experimental medicine research in mood and anxiety disorders.

**Methods/design:**

Predicting outcome following psychological therapy in IAPT (PROMPT) is a naturalistic observational project that began patient recruitment in January 2014. The project is currently taking place in Southwark Psychological Therapies Service, an Improving Access to Psychological Therapies (IAPT) service currently provided by the South London and Maudsley NHS Foundation Trust (SLaM). However, the aim is to roll-out the project across other IAPT services. Participants are approached before beginning treatment and offered a baseline interview whilst they are waiting for therapy to begin. This allows us to test for relationships between predictor variables and patient outcome measures. At the baseline interview, participants complete a diagnostic interview; are asked to give blood and hair samples for relevant biomarkers, and complete psychological and social questionnaire measures. Participants then complete their psychological therapy as offered by Southwark Psychological Therapies Service. Response to psychological therapy will be measured using standard IAPT outcome data, which are routinely collected at each appointment.

**Discussion:**

This project addresses a need to understand treatment response rates in primary care psychological therapy services for those with depression and/or anxiety. Measurement of a range of predictor variables allows for the detection of bio-psycho-social factors which may be relevant for treatment outcome. This will enable future clinical decision making to be based on the individual needs of the patient in an evidence-based manner. Moreover, the identification of individuals who fail to improve following therapy delivered by IAPT services could be utilised for the development of novel interventions.

## Background

Depression and anxiety within the general population are a continuing and significant public health problem [1]. The Adult Psychiatric Morbidity Survey showed that 16% of adults overall met the criteria for at least one common mental health disorder (defined in this survey as depression and/or anxiety disorders) [2]. Suffering from depression and/or anxiety has a significant impact on an individual’s life, including increased mortality [3], reduced quality of life [4] and reduced attendance and productivity at work [5]. The Improving Access to Psychological Therapies (IAPT) service was developed to provide psychological treatment for people with these common mental health disorders in order to address the high prevalence and burden of these disorders, and the problem that many people are untreated.

The therapeutic approaches used by IAPT are recommended as first-line treatment for mild to moderate depression and anxiety, in some cases along with appropriate medication, by the National Institute for Clinical Excellence [6]. IAPT services use a standardised protocol and collect considerable amounts of outcome data. This provides an opportunity for researchers to collect data on large populations (3000 per year in some services) of people with common mental health disorders undergoing similar treatments. Recently, the IAPT programme has reported early successes over its first three years, notably the treatment of “the first million patients” [7]. Overall recovery rates, defined as moving from caseness to non-caseness on measures of low mood and anxiety, were 45% in the last quarter of 2011/12, demonstrating consistent improvement over the duration of the programme and progression towards the original target of 50% [7].

Whilst these recovery rates are encouraging, they also show that around half of patients are not meeting standard definitions of recovery at the end of their treatments. Further, it is likely that a substantial proportion of those who do recover then go on to relapse in due course. Our knowledge of predictors of treatment response for depression and anxiety, both in terms of psychological and pharmacological treatments, is limited. It is likely that depression and anxiety disorders have many and varied causes, across psychological, social, and biological factors. Only by studying large cohorts of patients receiving treatments will it be possible to identify factors that predict positive or negative response and, by understanding these, develop new targets for treatment.

The aim of this project is therefore to provide an infrastructure to understand the predictors of treatment response, and to allow recruitment and follow up of subgroups of participants who do not respond to treatment in order to devise experimental studies to identify new treatments (both psychological and pharmacological). The project is supported by the SLaM Biomedical Research Centre which aims to provide infrastructure support for experimental medicine studies, and phase 1 and 2 trials.

### Project objectives

The objectives of the project are to:

1. Explore and describe the population attending for psychological therapy in one South London IAPT service (Southwark)

2. Embed data collection procedures within SLaM IAPT services to facilitate future research programmes

3. Collect biological samples to establish predictors of response to psychological therapy

4. Establish longitudinal predictors of response to psychological therapy

5. Identify participants for further, associated studies under the PROMPT theme

## Methods/design

### Design

This project uses a naturalistic, observational design. All eligible patients referred to Southwark Psychological Therapies Service are initially asked to consent to be contacted for research purposes, and those that agree are approached to take part in the project. Patients are approached before beginning therapy to allow collection of baseline measures and will then continue their treatment as usual within the IAPT service.

The project team carried out consultation with service users in the design of relevant materials. Firstly, the Southwark IAPT Service User Forum was consulted in two meetings about the procedure and suggested materials to be used in the project. Secondly, the FAST-R service (Feasibility and Support to Timely recruitment to Research) provided feedback and comments about the participant information sheet and consent forms used in this project.

### Ethical approval

The project has been approved by Bromley NHS Research Ethics Committee on 21^st^ October 2013 (reference: 13/LO/1347).

### Participants

Participants are recruited from one IAPT service provided by South London and Maudsley NHS Foundation Trust (SLaM). Inclusion criteria for this project are that patients are accepted by the IAPT service for treatment, and that they are able to give informed consent. Patients are excluded if they are not sufficiently fluent in English.

### Sample size

This is a naturalistic, observational project and therefore our sample size estimates are based on patient throughput and human resources. Based on these factors, we estimate to recruit up to 600 patients at baseline in the first year of the project. Using existing drop-out rates from SLaM IAPT services, we expect a drop-out rate of approximately 28%, leaving us with a sample of 472. According to power calculations, with power set at 0.8 and alpha at 0.05, this will allow us to detect standardised effect sizes of 0.13 in linear regression models estimating the effects of baseline factors in recovery. This is considered to be clinically significant; for example, a recent study of predictors of recovery [8] in the same setting showed that baseline PHQ-9 and GAD-7 scores were independently associated with recovery rates in a multiple regression model, with effect sizes ranging 0.14-0.21.

### Procedure

All data are collected at a baseline research visit, prior to the patients starting treatment in the service. These visits take place at the NIHR/Wellcome Trust King’s Clinical Research Facility. This visit involves a diagnostic interview carried out with a trained researcher, collection of biological samples (hair and blood), and completion of a range of questionnaires. Outcome measures will be collected in everyday contact with IAPT staff as part of routine IAPT practice at each treatment session and will be available on the IAPT patient record system. These variables are described below.

### Measures

#### ***Predictor variables***

The final choice of clinical and psychological predictors was based on discussions over a series of meetings with a large multidisciplinary group of academic experts, a systematic review of the literature conducted by the project coordinator (NG) and discussion over 3 subsequent meetings with the core Steering Committee who are all co-authors of this paper. The final list, which was reached by group consensus, has been considered comprehensive while at the same time manageable within a target completion time of 90 minutes for the whole assessment. This was judged to be the maximum time that we could impose on participants. Pilot work with the interview has confirmed that this is a realistic estimate of the time needed for completion.

PROMPT is collecting biological data as part of the National Institute for Health Research (NIHR) BioResource for Mental Health, which aims to enrol and collect biological samples from 52,000 healthy volunteers, patients and relatives. For PROMPT, this includes blood processed for DNA, RNA, buffy coat, serum and plasma. This sampling protocol allows for testing of an extensive battery of biomarkers relevant to treatment response in psychiatric disorders, and which are being routinely tested in a number of ongoing studies on a range of diagnostic categories. The BioResource project has separate ethical approval for the collection, storage and analysis of biological samples.

Demographic and patient characteristics are shown in Table 1. Table 2 shows the predictor variables, which will be collected at the baseline interview.

**Table 1:** Demographic and treatment factors

**Demographics**	
Socio-economic status	Current medication
Ethnicity	Physical health
Age	Benefits status
Working status	Marital status

**Table 2:** Predictor variables

**Predictors**	**Measure**	**Reference**
Biological sampling	Blood, saliva, hair	N/A
Diagnosis	MINI	[9]
Personality disorder	SAPAS	[10]
	SCID-II	[11]
Hypomania	Hypomania Checklist	[12]
Previous treatments and psychiatric history	Maudsley Staging Model	[13]
Social support	Oslo 3 Social Support Scale	[14]
Self-criticism	Forms of Self Criticising and Self Reassuring Scale (FSCRS)	[15]
Stressful life events	List of Threatening Experiences	[16]
Childhood abuse	Childhood Trauma Questionnaire	[17]
Quality of life	EQ-5D	[18]
Illness perceptions	Brief Illness Perceptions Questionnaire	[19]
Self-efficacy	General Self-efficacy Scale	[20]
Alcohol	AUDIT	[21]
Drug use	Drug abuse/dependence subscale - PDSQ	[22]

#### ***Diagnosis***

The Mini International Neuropsychiatric Interview (MINI) is used to assess diagnosis of participants in this project. The MINI is a structured interview which assesses DSM diagnoses, and is rapid to administer. The MINI covers the following diagnoses: Major Depressive Episode (MDE); recurrent MDE; MDE with melancholic features; dysthymia; suicidality; mania and hypomania; panic disorder; social phobia; agoraphobia; obsessive compulsive disorder; post traumatic stress disorder; alcohol abuse; alcohol dependence; substance use; substance dependence; psychotic disorders; mood disorder with psychotic features; anorexia; bulimia; generalised anxiety disorder; and antisocial personality disorder. The MINI has good internal reliability and consistency [9].

#### ***Personality disorder***

All participants complete the Standardised Assessment of Personality – Abbreviated Scale (SAPAS) [10], an eight item screen for personality disorder. Each item is rated as yes or no, giving a maximum score of eight. In clinical populations, a cut-off three is suggested for identification of cases at high risk of personality disorder. The SAPAS has acceptable internal consistency (Cronbach’s alpha = 0.68). Participants will also complete the borderline personality subsection of the Structured Clinical Interview for DSM-IV Personality Disorders (SCID-II; [11]). The SCID-II is a semi-structured interview delivered by a trained member of the research team to assess presence of anti-social personality disorder and borderline personality disorder. Questions are rated as 1 “absent”, 2 “sub-threshold” or 3 “threshold”. The SCID-II demonstrated acceptable test-retest reliability (kappa = 0.68) as well as acceptable inter-rater reliability (kappa = 0.68) in previous studies.

#### ***Hypomanic symptoms***

The Hypomania Checklist (HCL-16) is used to screen for the presence of hypomanic symptoms to aid in the diagnosis of bipolar disorders [12]. The HCL-16 has been developed from the original HCL-32 and studies have supported the reliability and validity of this shorter measure [12]. Each item is rated as either “yes, present or typical of me” or “no, not present or not typical of me”. There is support for a two factor structure for this scale, the first a ‘active-elated’ factor and the second a ‘risk-taking/irritable’ factor. Reliability for these two subscales and the total score are within acceptable levels (Cronbach’s alphas 0.65-0.77).

#### ***Previous treatments***

Information will be collected about the number of previous episodes each patient has experienced, duration of previous episodes, their previous history of psychological or pharmacological treatment and the age of onset. Current prescribed medication will also be recorded, including type of drug, number of doses per day and dosage. Treatment resistance will be quantified using a modified version of the Maudsley Staging Model [13].

#### ***Social support***

Social support is measured using the three-item Oslo Social Support scale (OSS-3) [14]. The first question measures how many people the participant is close to, with response options ranging from none to more than five. The second question asks the participant to rate how much of an interest other people take in the participant’s life. The third question asks participants to rate if they could get practical help from their neighbours, with responses ranging from very easy to very difficult.

#### ***Self-critical thoughts***

Participants complete the Forms of Self-critical/Attacking and Self-reassuring Scale (FSCRS) [15]. This is a 23-item measure eliciting a score for self critical thinking and self-reassuring thoughts. The self-critical subscale is divided in to hated self, inadequate self, and reassured self. Items are rated using a Likert scale ranging from 0 “not at all like me” to 4 “extremely like me”. The three subscales have good internal consistency with Cronbach’s alphas of 0.86-0.90 [15]. A recent study has supported the three factor structure of this scale [23].

#### ***Life events***

Stressful life events are assessed using the List of Threatening Events Questionnaire [16], a 12-item measure which includes negative events such as serious illness, death, unemployment and loss of an important relationship. Participants mark whether the event has happened to them, and if it has, the date that it occurred. The scale has good test-retest reliability for most items (kappa = 0.78 – 1.0) with the exception of the question relating to having an item stolen, which has lower reliability (kappa = 0.24). There is also good agreement between individual participants and others (kappa = 0.7 - 0.9) and good agreement with interview based measures (sensitivity = 89%, specificity = 74%).

#### ***Childhood trauma***

Traumatic events during childhood are measured using the Childhood Trauma Questionnaire, a 28-item measure that covers five domains. These are: emotional abuse, physical abuse, sexual abuse, emotional neglect, and physical neglect. Questions include “people in my family hit me so hard that it left bruises or marks” (emotional abuse). Each item is rated on a five point Likert scale from “never true” to “very often true”, with scores for each sub scale ranging from 5-25, and the total score from 25-125. The subscales have good internal consistency with Cronbach’s alphas ranging from 0.61 – 0.92) [17].

#### ***Quality of life***

Quality of life is measured using the EuroQol (EQ-5D) [18] which consists of a five part questionnaire and one visual analogue rating scale. The five questions cover self-care, pain, mood, mobility and usual activities and are answered on a three-point Likert scale, ranging from no problems to extreme problems. The visual analogue scale asks participants to indicate how they would rate their health overall today, on a scale from 0 “worst health imaginable” to 100 “best health imaginable”.

#### ***Illness perceptions***

Beliefs about illness are assessed using the nine-item Brief Illness Perceptions Questionnaire [19]. Five questions assess cognitive representations of illness, two assess emotional representations and one assesses illness comprehension. These questions are rated from 0 to 10. The final question asks respondents to list three factors they consider had a causal role in their illness. The original questionnaire contains the term “illness” but this has been changed to “depression/anxiety” for the current project.

#### ***Self-efficacy***

Self-efficacy is measured using the General Self-efficacy Scale [20]. This is a ten item measure which is rated from 1 “not at all” to 4 “exactly true” and is designed to assess respondents beliefs in their ability to complete tasks. The scale has been shown to have good reliability across a variety of participant groups (Cronbach’s alpha ranging from 0.76-0.90).

#### ***Alcohol***

Alcohol consumption is assessed using the 10-item Alcohol Use Disorders Identification Test (AUDIT). The questions in this scale measure alcohol consumption, dependence and problems related to alcohol use. For example, questions include “How often during the last year have you failed to do something that was expected of you because of drinking?” Items have different response categories but each are scored from 0 – 4, giving a possible range of 0 – 40. A score of above eight is indicative of a problem with drinking [21]. A review paper including 18 studies using the AUDIT reported that internal consistency is reliably reported to be above 0.80 [24].

#### ***Drug use***

Drug use is assessed using the drug use/dependency subscale of the Patient Diagnostic Screening Questionnaire [22]. This is a six item measure with response options of yes or no. Questions include “(during the past two weeks) did you think you had a drug problem?”.

#### ***Outcome measures***

The primary outcome for PROMPT will be symptom change for depression and anxiety, as measured nationally by IAPT using by the PHQ-9 (Patient Health Questionnaire) and GAD-7 (Generalised Anxiety Disorder assessment) [25,26]. The PHQ-9 is a self-administered scale covering nine symptoms of depression, which are rated from 0 “not at all” to 3 “nearly every day”. With a sample of patients from primary care, the scale was found to have good reliability (Cronbach’s alpha = 0.89) and sensitivity and specificity of 88% [25]. The GAD-7 is a seven item self report measure, rated from 0 “not at all” to 3 “nearly every day”. The scale has good reliability (Cronbach’s alpha = 0.92) and sensitivity of 89% and specificity of 82% [26].

Both these measures are routinely collected as part of standard IAPT practice at every treatment session. Data will be gathered from the IAPT patient records system (IAPTus) with prior consent of participants. A score of 10 or more on the PHQ-9, and a score of 8 or more on the GAD-7 indicates caseness [25,26]. Participants who score above these thresholds for caseness on both the PHQ-9 and GAD-7 at their final appointment, will be defined as being in the ‘non-recovered’ group. The standard IAPT measure of Reliable Change Index will also be used as an outcome measure of treatment response which will be based on a drop of 6 points for the PHQ-9 and a drop of 5 points for the GAD-7 [27].

Secondary outcomes will be employment status, benefits status, engagement with services, and work and social functioning. Employment and benefits data is routinely collected at each IAPT appointment and entered on to the patient records system. Measures of engagement with IAPT services will include failure to engage, drop-out or discharge. Work and social functioning is measured using the Work and Social Adjustment Scale, a five item self-report measure collected as part of the routine IAPT outcome measures. This information will be collected from the care pathway of the patient records system IAPTus, which is routinely updated by IAPT clinical and admin staff.

### Analysis

Descriptive summaries of socio-demographic information will be provided for baseline, and follow up time-points for the sample as appropriate. Descriptive summaries at the level of IAPT services will also be provided. Missing data will be reported for all treatment time-points to describe retention/drop-out rates. Reasons for drop-out will be recorded. We will use the STROBE statement (http://www.strobe-statement.org/index.php?id = available-checklists) for a list of items to be reported. All statistical analyses will be carried out in the statistical software package STATA. Descriptive summaries for all outcome measures will be provided. Continuous outcome measures will be inspected visually to assess the normality of data and to check for outliers. Univariate associations with IAPT non-recovery will be explored using t-tests (for normally distributed data) and Mann-Whitney tests (for skewed data). The independence of associations between the proposed predictor variables and IAPT non-response will be explored using linear mixed model methods (for continuous outcome measures) and logistic regression (for categorically-defined outcomes i.e. recovered vs. non-recovered). The possibility of sex interactions and non-linear effects will be examined. Analysis of residuals from the model will be performed to assess the model fit and to check for outliers.

## Discussion

The project outlined in this paper aims to identify factors that predict response to psychological therapy in a primary care setting. Psychological therapy for depression and anxiety is currently recommended as a first-line therapy by clinical guidelines [6], yet is currently only available to 10-15% of sufferers [7]. Better targeted services are needed as access is unlikely to increase significantly and it is also currently uncertain to what degree the new Clinical Commissioning Groups will fund IAPT schemes. Outcome data currently available suggest that over half of patients are not meeting nationally recognised recovery rates [8]. At present there have been no other large-scale, naturalistic studies investigating predictors of outcome in an IAPT setting. The IAPT setting is particularly applicable to research, as patients are required to complete standard measures of mood and functioning at each contact. This project aims to embed recruitment practices in to an IAPT service allowing further studies to be carried out in the future. A secondary aim of this project is be extend the research to other IAPT sites.

Participants will complete therapy in a real world clinical setting allowing collection of meaningful observational data. Participants will be interviewed before beginning treatment, thus allowing collection of baseline data which can be used to predict future treatment outcome. Because of the nature of the outcome measurement in IAPT, participants will not need to attend for a second research interview as this information can be collected from the IAPT electronic patient records system. The numbers of patients attending for psychological treatment at IAPT services has been steadily increasing, such that, this is now the commonest route of talking therapy for mild to moderately affected people. A large scale research study investigating predictors of treatment response in this setting is therefore essential.

Collecting data from participants in a naturalistic setting, whilst providing ecologically valid data, is not without its limitations. We can anticipate around 30% drop-out of patients during IAPT therapy, meaning that we will lose some final outcome data for many patients we see at baseline. However, as data is collected on every contact, with a national completion rate of 90%, this will be minimised as we will be able to use data from the final session attended. This also affords us the opportunity to investigate if there are common factors that may explain drop-out. IAPT therapy is not standardised and this will introduce variability within our sample. Patients may receive one-to-one cognitive behavioural therapy with a trained CBT therapist, or a clinical psychologist. They may also receive guided self-help with a psychological well-being practitioner. Further, patients may attend for group therapy or psycho-educational workshops. Increasingly, IAPT services are offering alternatives to CBT including but not limited to; interpersonal psychotherapy, couples therapy, and eye movement desensitization and reprocessing therapy.

IAPT services are accessed by a wide variety of patients, with a diverse range of presenting complaints. The project will initially focus its recruitment on one borough (Southwark) whose services are currently provided by South London and Maudsley NHS Foundation Trust. In terms of ethnic diversity, the Office for National Statistics reported that the population of Southwark comprises 62% from White groups, 27% from Black groups and 11% from other groups. There will also be variety in use of prescribed medication amongst the participants, and this information is not always routinely collected within IAPT services. To overcome this, participants will be asked about their prescribed medication at the research interview. However, it will not be possible to monitor any changes in dosage or medication when looking at outcomes following treatment. As part of the standard IAPT data collection, participants are asked if they are prescribed medication, and if they are currently taking their medication. It is possible that a participant may change dose or even drug whilst receiving therapy.

Whilst one of the strengths of this project is its naturalistic nature, this also presents some challenges in terms of the analysis of outcome data. In large scale randomised controlled trials that compare efficacy of different therapies, or medication vs therapy, manualised therapy is often used. Therapists in such studies will be supervised more closely than in standard practice, will be less free to vary sessions and will have their skills monitored. This project will involve therapists from different backgrounds including but not limited to; trained CBT therapists; clinical psychologists with IAPT high intensity therapy training and psychological well-being practitioners both pre- and post-qualification. These therapists will also have different amounts of experience and bring their own styles and ways of working to their patients. All of this will contribute to the variance in non-specific therapeutic factors influencing outcomes. At this point, we have not identified an efficient way of measuring these important ‘therapist factors’ but we will be working with team leaders in order to capture this in future research.

It is possible that patients who agree to take part in the project may not be representative of the IAPT population as a whole. Data will be available for participants who do not consent to take part in the main research program so it will be possible to compare participants with non-participants on standard factors such as demographics and initial PHQ-9 and GAD-7 scores. However, it is possible that other factors may prevent some people from agreeing to take part in the research such as health literacy, previous experience of psychological therapy and employment status. Due to the variability both in the participants, the therapists and also the therapy offered, the project will rely on a large sample size to detect clinically significant effects.

## Conclusions

PROMPT aims to identify predictors of non-response to first line psychological treatment provided by at an IAPT service within a diverse population of South London. The aim is to translate findings into improved treatment outcomes, better patient care and more efficient services within IAPT, and to support ongoing research into the causes and effective treatment of IAPT non-responders.

## Competing interests

The authors declare that they have no competing interests.

## Authors’ contributions

NG was the project manager and responsible for the initial draft of this manuscript. GB, AC, and CMP contributed to the statistical design and the biological data collection methodology. NH is currently responsible for the project management, and SK for collecting data. All authors contributed to the development of the protocol, commented on the initial draft and approved the final version of the manuscript.

## Pre-publication history

The pre-publication history for this paper can be accessed here:

http://www.biomedcentral.com/1471-244X/14/170/prepub
